# Supplementary material for: User preferences for an mHealth app to support HIV testing and pre-exposure prophylaxis uptake among men who have sex with men in Malaysia
Source: PLOS Digit Health. 2024 Oct 30;3(10):e0000643. doi: 10.1371/journal.pdig.0000643 (PMC11524455; doi:10.1371/journal.pdig.0000643)
Supplement: Attachment — Submitted filename: Response to Reviewers.docx [file pdig.0000643.s001.docx]

Dear Dr. Leo Anthony Celi

We thank you for your continued consideration of our manuscript, “User Preferences for an mHealth App to Support HIV Testing and pre-exposure prophylaxis Uptake among Men Who Have Sex with Men in Malaysia.” The reviewers’ comments strengthened the paper in both clarity and content. We have revised our manuscript to include the additional details requested by the reviewers, and it is much improved as a result.

Please see our responses to your and the reviewers’ comments below. As instructed, changes to the manuscript have been made using tracked changes.

We appreciate the opportunity to resubmit this manuscript for your consideration.

Sincerely,
Roman Shrestha

_________________________________________________________

**Reviewer #1 Comments and Responses:**

1. Introduction: please include a more detailed discussion on existing mHealth interventions globally and their impact, setting the context for why this study is necessary.

**Response: We have added additional detail to the introduction to provide a broader context for the study.**

2. The abstract is comprehensive, but it is somewhat lengthy. Consider condensing it to ensure it succinctly captures the key points without overwhelming the reader.

**Response: We have condensed the abstract to make it more concise.**

3. Methods: The recruitment process is well-explained, but more details on how participants were selected and any potential biases this might introduce would be beneficial. For instance, did recruitment through social media platforms like Grindr and Hornet influence the sample's demographic characteristics?

**Response: We have included more about how this may have influenced our data as a limitation. Specifically, this may have yielded a sample that has more positive feelings and comfort using technology for their health needs.**

4. In the data analysis section, please explain the coding process and how themes were identified and validated.

**Response: We have added further details about the final themes and the coding process used to identify them.**

5. Results: The results section is thorough, presenting detailed preferences and concerns of MSM and stakeholders. However, it would be helpful to include more direct quotes from participants to vividly illustrate the themes.

**Response: Response: We have added more direct quotes from participants to enhance the illustration of the themes.**

6. Table 1 and Table 2 provide helpful information but could be more effectively integrated into the text. Discussing these tables in more detail within the narrative would enhance their relevance.

**Response: We appreciate the reviewers' feedback. While we do agree that integrating this information might enhance the narrative, we believe that the table provides the information in a concise manner that is easy for the reader to digest.**

7. Discussion: Please strengthen this section by linking the results more explicitly to the existing literature on mHealth interventions for HIV prevention. This would help to situate the findings within the broader field.

**Response: We have strengthened the discussion by incorporating more explicit connections to the broader field of research on mHealth interventions for HIV prevention.**

8. Please discuss on the potential impact of these limitations in more detail. For example, how might the recruitment method have biased the findings?

**Response: We have expanded the limitations section to discuss the potential impact of the recruitment method on the findings in more detail.**

9. Please expand the section on the conclusion to provide more specific recommendations for future research and practical implementation of the app.

**Response: We have added specific recommendations to the conclusion.**

10. It would be valuable if you could elaborate more on how ethical concerns, especially regarding confidentiality and data security, were addressed during the study.

**Response: We have included additional information on how privacy and confidentiality were addressed during the study.**

11. The manuscript presents significant findings that can contribute to the development of an effective mHealth app for HIV prevention among MSM in Malaysia.
    **Response: Thank you very much for the positive feedback.**

**Reviewer #2 Comments and Responses:**

1. The manuscript meet PLOS Digital Health’s publication criteria and is technically sound, and the data support the conclusions. The manuscript describes methodologically and ethically rigorous research with conclusions that are appropriately drawn based on the data presented.

**Response: Thank you so much for your positive evaluation and feedback on our manuscript.**

2. The statistical analysis has been performed appropriately and rigorously

**Response: Thank you for acknowledging the rigor of our analysis**.

3. Authors made all data underlying the findings in their manuscript fully available

**Response: Thank you for your feedback.**

4. The manuscript is presented in an intelligible fashion and written in standard English with minimal grammatical errors that need to be corrected by the author.

**Response: We have reviewed the manuscript to correct any grammatical errors.**

5. In the Methods section, you mentioned that there were 36 participants in the study, specifically 20 MSM and 16 community stakeholders. However, you also mention clinical stakeholders later on. Were the clinical stakeholders a subset of the 16 community stakeholders? Please clarify this point.

**Response: Thank you for your careful review and for pointing out the need for clarification. To clarify, the stakeholders were individuals who met the following inclusion criteria: 1) age 18 years or older; 2) currently employed as a physician, counselor, pharmacist, or as NGO staff involved in providing HIV prevention and treatment services to MSM. Thus, clinical stakeholders, such as physicians, counselors, and pharmacists, were indeed a subset of the 16 community stakeholders. We have revised the methods section to explicitly reflect the inclusion criteria of stakeholders to minimize any confusion.**

6. What did you mean by this statement "To be eligible, community and clinical stakeholders must have been involved with providing HIV related services to MSM"? Please improve the write up under participants and settings section.

**Response: Thank you for this suggestion. We have clarified the eligibility criteria in the revised manuscript.**

7. Based on your methodology, it appears that this was a mixed methods study design. However, this is not clearly articulated in your write-up. Please review this section to clearly include and describe the study design used for this study.

**Response: The current work is a qualitative study. We included only collected demographic information in a quantitative format for descriptive purposes.**

8. I noticed that you referred to the COVID-10 pandemic in the discussion section, which is incorrect. Please review and update it to reflect the correct year.

**Response: We have corrected this typographical error in the revised manuscript**
